# Pride-based violence, intoxicated sex and poly-drug use: a vocational school-based study of heterosexual and LGBT students in Bangkok

**DOI:** 10.1186/s12888-022-03777-7

**Published:** 2022-02-24

**Authors:** Yamol Kongjareon, Nattharat Samoh, Pimnara Peerawaranun, Thomas E. Guadamuz

**Affiliations:** 1 Center of Excellence in Research on Gender, Sexuality and Health, Faculty of Social Sciences and Humanities, Mahidol University, 999 Phuttamonthon 4 Road Salaya, Nakhon Pathom, 73170 Thailand; 2 Department of Society and Health, Faculty of Social Sciences and Humanities, Mahidol University, Nakhon Pathom, Thailand

**Keywords:** Poly-drug use, Vocational school students, Violence, Intoxicated sex, LGBT, Thailand

## Abstract

**Background:**

In Thailand, drug use is widely observed, especially among vocational-school students, who are more inclined to use various types of drugs and to experience pride-based violence (violence based on institutional pride, honor, or on seniority status) than any other groups of students. Drug use contexts differ based on sexual orientation and gender identity (e.g., clubs). This study aims to examine the prevalence and correlates of poly-drug use (the use of at least three types of drugs) among vocational-school students, with a focus on sexual orientation and gender identity.

**Methods:**

In this study, 638 vocational school students living in Bangkok metropolitan area participated in a three-year longitudinal survey of four vocational schools. Experiences of violence, sexual behaviours, and poly-drug use were assessed and analyzed using multivariable logistic regression.

**Results:**

About one tenth of all students (11.3%) reported poly-drug use, a fifth among Lesbian, Gay, Bisexual and Transgender (LGBT) students (20.2%) and almost a tenth of heterosexual students (9.8%). In the multivariable logistic model of LGBT students, GPA, pride-based violence, intoxicated sex, and counseling needs were significantly associated with poly-drug use (AOR = 4.62; 95% CI 1.17–18.29, AOR = 6.01; 95% CI 1.31–27.32, AOR = 5.17; 95% CI 1.10–24.28, AOR = 4.64; 95% CI 1.16–18.54, respectively). Likewise, among heterosexual students, GPA and intoxicated sex were significantly associated with poly-drug use (AOR = 2.02; 95% CI 1.09–3.75, AOR = 5.31; 95% CI 2.81–10.04, respectively).

**Conclusions:**

LGBT vocational-school students have significantly higher prevalence of poly-drug use than their heterosexual peers. Correlates include lower GPA, having experienced pride-based violence and intoxicated sex. School-based intervention programs should also address pride-based violence and intoxicated sex in their harm reduction programs.

## Background

Drug problems are currently widespread across the world. According to the *World Drug Report 2018* [1], the worst drug situation is among Southeast Asian countries, partly due to their proximity to the largest drug production and trafficking area, commonly known as the Golden Triangle. Thailand is one of the countries that has an ongoing drug problem, especially among youths. At present there are a lot of drugs being imported into the country, which results in greater supply than drug users’ demands, making drugs affordable for youths [2]. Of the Thai population aged between 15 and 24 years, 39% are involved with illicit drugs (e.g., ever use amphetamine, crystal methamphetamine, marijuana, heroin, ketamine) [2]. There are about 2.7 million youths using drugs in Thailand. Among youths aged between 15 and 19 years, there are about 300,000 who have needs for drug treatment [3].

Most of Thai youths involved with drugs are vocational-school students, which nearly doubles in number compared to secondary-school students. Vocational-school students tend to have easier access to drugs and high-risk behaviours based on the sense of pride, institutional dignity, masculinity, and brotherhood of students within the same institution [4–9]. Vocational schools in Thailand are like trades schools or technical schools where students learn various skills like accounting, auto mechanics and electronics. Youngest students are about 16 years old whereby they matriculate after graduating from the nineth grade. Instead of attending the tenth grade in a secondary school, they can attend a vocational school instead and receive an associate degree. A previous study of vocational school students’ drugs and alcohol behaviour patterns found that most students use more than one type of drugs. “Sociality” is found to be one of the key factors that introduce vocational school students to drugs by way of new groups of friends or new social environments [10]. Adolescents often seek social and peer support in entering a new environment [11]. Drug use thus becomes an instrument they use for gaining acceptance from their upperclassmen or new friends. Drug use can also be a tool to cultivate power and status. Drug users are sometimes perceived as wealthy and capable of controlling themselves. Once they gain power, they gain acceptance and respect [12–14].

Poly-drug use, however, can end up bringing various health risks, including substance use disorders and fatal overdoses [15]. What’s more, studying in vocational schools is not valued in Thai society since vocational school students are perceived as mischievous, lazy, unscholarly and they tend to be from children of low-income earners/working class families. Vocational school students thus become labeled as part of social problems, and that makes them vulnerable [16].

Previous studies among heterosexual adolescents have not found a relationship between sex at birth and drug use. However, drug use among LGBT adolescents, particularly among men who have sex with men (MSM), have been associated with high-risk sexual activities and violence through the syndemics theory which posits that psychosocial conditions like substance use, violence, depression, and unprotected sex interact and amplify one another which may lead to higher risks for HIV acquisition [17, 18]. Since many vocational school students are using drugs, poly-drug use among them may be common. To our knowledge, however, there has not been a study on poly-drug use, comparing LGBT and non-LGBT vocational school students. This research thus aims to study factors associated with poly-drug use among LGBT and non-LGBT vocational school students in Bangkok.

## Methods

### Sample

This study included 638 vocational first-year (equivalent to grade 10) students from four vocational schools in Bangkok, two private and two public schools. An online questionnaire was used that participants accessed through desktop computers, tablets, and smartphones. Mahidol University Social Sciences Institutional Review Board (MUSSIRB) reviewed and approved the study (Certificate of Approval No. 2017/161.2607). For participants younger than 18 years, MUSSIRB approved waivers of parental permission, informed by our previous study on proxy permission and waivers of parental permission [19]. Before enrollment into the study, we provided a detailed explanation of the study’s risks and benefits to the students. All students provided assents/consents to participate verbally with research staff and then again online by clicking through the assent/consent process. All participants were also given a participant information sheet to take home that detailed the study’s risks and benefits and contained contact information of the study staff and of the MUSSIRB Chairperson. Participation in this study was voluntary and anonymous.

### Measures

Demographic characteristics/general information and other variables included gender and sexual orientation which used different questions including (Note: texts in quotes are translated from the Thai questionnaire): “Which of these words do you think describes you best?” and “If you were to have a committed partner (*faen*), which gender would you like them to be?” Choices included man, woman, ladyboy/transgender women, gay men/MSM, tomboy, transgender men, lesbians and bisexuals. There were also questions about age, educational institution, Grade Point Average (GPA), and some sensitive behavioral questions like “Have you experienced violence based on institutional pride, honor, or seniority status?” Choices included yes, both perpetrator and victim, or either one/never). “Have you used drugs (Crystal Meth, Ketamine, Ecstasy) before or during sexual activities.” Choices included often/sometimes or never, and “Do you need advice in sensitive subjects such as problems regarding your lifestyle, emotions, feelings, etc.” Choices included yes I do, or no I don’t.

Poly-drug use is measured through usage of at least three types of drugs [20]. These questions include “Have you used the following substances?” informed by our previous research and the literature [21, 22], options included kratom cocktail/4 × 100, pro, B5, Xanax/Alprazolam, amphetamine, crystal methamphetamine, ecstasy/MDMA, ketamine, injecting heroin and injecting crystal methamphetamine. Choices included Yes in the past one month, Yes in the past three months, Yes in the past six months, Yes in the past one year and Yes more than one year ago or Never. If their answer is Yes in past one month, Yes past three months or Yes past six months, they will be considered having experienced using that drug [22].

### Data analysis

Statistics used in this analysis included descriptive statistics, Pearson’s *χ*^2^ test, binary and multivariable logistic regression analyses where poly-drug use is the dependent variable, and where education are adjusted variables. GPA, pride-based violence, drug use before or during sex, and counseling needs were independent variables. STATA 14.0 software (StataCorp, Texas, U.S.A.) were used for all statistical analyses.

## Results

Table 1 shows demographic and behavioral characteristics of 638 vocational school students in Bangkok who provided consent, met the eligibility criteria, and completed the online questionnaire. Of participants, 549 identified as heterosexual male/heterosexual female (86.1%) and 89 identified as LGBT (13.9%). Among all participants, 337 were below age 16 years (52.8%), 334 (52.4%) were studying in public schools, and 380 (59.6%) had their GPA above 2.5. Moreover, 107 (16.8%) had experienced pride-based violence, 131 (20.5%) had used drugs before or during sex, and 153 (24.0%) needed counseling. More than one-tenth (72 students, 11.3%) used multiple substances. Among them, 18 students identified as LGBT (25.0%), while 54 identified as heterosexual male/heterosexual female (75.0%).

**Table 1:** Demographic and behavioral characteristics of vocational school students in Bangkok (*N* = 638)

	Total (***N*** = 638)	LGBT (***n*** = 89)	Heterosexual male/female (***n*** = 549)	***p***-value^***d***^
**Age**				
Less than or equal to 16 years	337 (52.8%)	52 (85.4%)	285 (51.9%)	0.253
More than or equal 17 years	301 (47.2%)	37 (41.6%)	264 (48.1%)	
**Type of School**				
Public	334 (52.4%)	53 (59.6%)	281 (51.2%)	0.143
Private	304 (47.6%)	36 (40.4%)	268 (48.8%)	
**GPA**^***a***^				
Less than or equal to 2.5	258 (40.4%)	33 (37.1%)	225 (41.0%)	0.486
More than 2.5	380 (59.6%)	56 (62.9%)	324 (59.0%)	
**Pride-based Violence**^***b***^				
Yes	107 (16.8%)	19 (21.3%)	88 (16.0%)	0.213
No	531 (83.2%)	70 (78.7%)	461 (84.0%)	
**Intoxicated sex**				
Yes	131 (20.5%)	16 (18.0%)	115 (21.0%)	0.520
No	507 (79.5%)	73 (82.0%)	434 (79.0%)	
**Counseling need**				
Yes	153 (24.0%)	29 (32.6%)	124 (22.6%)	0.040
No	485 (76.0%)	60 (67.4%)	425 (77.4%)	
**Substance use**				
Kratom cocktail/4 × 100	166 (26.0%)	22 (24.7%)	144 (26.2%)	0.763
Pro	150 (23.5%)	22 (24.7%)	128 (23.3%)	0.772
B5	50 (7.8%)	12 (13.5%)	38 (6.9%)	0.033
Xanax / Alprazolam	37 (5.8%)	17 (19.1%)	20 (3.6%)	< 0.001
Amphetamine (*Yaba/Yama*)	23 (3.6%)	8 (9.0%)	15 (2.7%)	0.003
Crystal Methamphetamine	26 (4.1%)	8 (9.0%)	18 (3.3%)	0.011
Ecstasy/MDMA (E)	26 (4.1%)	10 (11.2%)	16 (2.9%)	< 0.001
Ketamine	60 (9.4%)	11 (12.4%)	49 (8.9%)	0.303
Injecting Heroin	16 (2.5%)	7 (7.9%)	9 (1.6%)	< 0.001
Injecting Crystal Methamphetamine	15 (2.4%)	7 (7.9%)	8 (1.5%)	< 0.001
**Use of more than one substance**				
Two or more	148 (23.2%)	24 (27.0%)	124 (22.6%)	0.364
Three or more / **Poly-drug use**^***c***^	72 (11.3%)	18 (20.2%)	54 (9.8%)	0.004

^*a*^ GPA, Grade Point Average, ranges from 0.0 to 4.0 with 4.0 representing a perfect academic record. We chose 2.5 as a cutoff point because in Thailand, one would need a minimum 2.5 GPA to qualify for admission to higher education

^*b*^ Pride-based violence is defined as violence based on institutional pride, honor, or seniority status

^*c*^ Poly-drug use refers to any three of the following drugs: kratom cocktail/4 × 100, pro, B5, Xanax/Alprazolam, amphetamine, crystal methamphetamine, ecstasy/MDMA, ketamine, injecting heroin and injecting crystal methamphetamine in part six months

^*d*^ *p*-value obtained from Pearson’s *χ*^2^ test

According to Models 1 and 2 in Table 2, having GPA equal to or less than 2.5 and having intoxicated sex were significantly associated with poly-drug use, regardless of identifying as LGBT or not. However, as shown in Table 2, having GPA equal to or less than 2.5 were significantly higher for LGBT identified students, as compared to heterosexual male/female students (AOR = 4.62; 95% CI 1.17–18.29 versus AOR = 2.02; 95% CI 1.09–3.75). Compared to heterosexual students, a smaller proportion of LGBT students engaged in intoxicated sex (AOR = 5.17; 95% CI 1.10–24.28 versus AOR = 5.31; 95% CI 2.81–10.04). Finally, having experienced pride-based violence and counseling needs were only significant among LGBT students, and not among heterosexual male/female students (AOR = 6.01; 95% CI 1.32–27.32 versus AOR = 1.03; 95% CI 0.50–2.13 and AOR = 4.64; 95% CI 1.16–18.54 versus AOR = 1.12; 95% CI 0.56–2.21).

**Table 2:** Factors associated with poly-drug use, classification of sexuality among vocational school students in Bangkok (*N* = 638)

	Model 1^***b***^				Model 2^***b***^			
	LGBT ***N*** = 89				Heterosexual male/female ***N*** = 549			
	Poly-drug use^***a***^ (***n*** = 18 (20.2%))				Poly-drug use^***a***^ (***n*** = 54 (9.8%))			
	OR	95% CI	AOR	95% CI	OR	95% CI	AOR	95% CI
**GPA**^***c***^								
Less than or equal to 2.5	4.76**	(1.58–14.37)	4.62*	(1.17 18.29)	2.71**	(1.51–4.84)	2.02*	(1.09–3.75)
More than 2.5	Ref.		Ref.		Ref.		Ref.	.
**Pride-based Violence**^***d***^								
Yes	8.61***	(2.69–27.56)	6.01*	(1.32–27.32)	2.47**	(1.31–4.67)	1.03	(0.50–2.13)
No	Ref.		Ref.		Ref.		Ref.	
**Intoxicated sex**								
Yes	9.14***	(2.73–30.64)	5.17*	(1.10–24.28)	6.59***	(3.66–11.88)	5.31***	(2.81–10.04)
No	Ref.		Ref.		Ref.		Ref.	
**Counseling needs**								
Yes	3.42*	(1.18–9.95)	4.64*	(1.16–18.54)	1.36	(0.72–2.56)	1.12	(0.56–2.21)
No	Ref.		Ref.		Ref.		Ref.	.

^*a*^ Poly-drug use refers to any three of the following drugs: kratom cocktail/4 × 100, pro, B5, Xanax/Alprazolam, amphetamine, crystal methamphetamine, ecstasy/MDMA, ketamine, injecting heroin and injecting crystal methamphetamine in part six months

^*b*^ All model adjusted for type of school

^*c*^ GPA, Grade Point Average, ranges from 0.0 to 4.0 with 4.0 representing a perfect academic record. We chose 2.5 as a cutoff point because in Thailand, one would need a minimum 2.5 GPA to qualify for admission to higher education

^*d*^ Pride-based violence is defined as violence based on institutional pride, honor, or seniority status

*p < = .05; **p < .01;***p < .001

## Discussion

About one-tenth of Bangkok vocational school students engage in poly-drug use (use of at least three types of drugs in the past six months). However, when stratified by LGBT status, the prevalence doubles for vocational school students who identify as LGBT (9.8% versus 20.2%). This significant disparity is also associated with a number of school-related factors such as having lower GPA, having experienced pride-based violence, and having experienced intoxicated sex.

Another significant finding from this study is that LGBT students are more likely than non-LGBT students to engage in poly-drug use. One study in the United States found that LGBT students were three times more likely to engage in poly-drug use, compared to heterosexual students [23]. To our knowledge, this association has not been explored in Thailand.

We also found that lower GPA, having experienced pride-based violence and using drugs before or during sex were significantly associated with poly-drug use. Additionally, these strengths of association were much higher among LGBT vocational school students, compared to non-LGBT vocational school students. A systematic review of polysubstance use among adolescents age 10–19 years found that polysubstance use was associated with poor academic performance [24]. A recent study conducted in Thailand also found an association between lower academic performance and polysubstance use [25]. The association between intoxicated sex and poly-drug use was found among gay, bisexual and other men who have sex with men in Dublin, Ireland. The study also found that group sex, pleasure and sexual confidence were significantly associated with poly-drug use [26].

As for pride-based violence, this seems to be a unique Thai cultural context. We did not find similar studies abroad. A study in Thailand has claimed that substance use was the cause of general violence [27]. The National Survey of Child and Adolescent Well-being (NSCAW II) study conducted between March 2008 and September 2009 found teens age 11–17 years who engaged in poly-drug use were six times more likely to have been victims of physical abuse, compared to teens who did not engage in poly-drug use [28].

In Thailand, vocational school students are likely to receive less opportunities than secondary school students. Moreover, LGBT groups are not as accepted by Thai society, despite foreigners believing that Thailand is a “gay paradise.” [29]. Furthermore, students who have lower GPA are usually less valued, compared to those with higher GPA. All this leads LGBT vocational school students to be marginalized and deprived of opportunities [29], which may indirectly force them to engage in drug use in order to cope with their problems. However, we also found that LGBT vocational school students want counseling and someone to talk to about their problems.

There are some limitations in this study. First, this study is only limited to vocational schools in Bangkok metropolitan area, and so findings might not be generalizable to all vocational schools in Thailand. However, this study is a first step into understanding poly-drug use in vocational schools. Second, we did not specifically assess for concurrent use in our survey; some participants may have used the drugs currently and others not. Certainly, future studies should include concurrent use and expand to other provinces and regions throughout Thailand, as well as conduct a longitudinal study to understand factors related to poly-drug use in order to inform preventive interventions. Thai vocational schools should have trained counselors for students who want to reach out and discuss trauma related to pride-based violence. There can also be informal outreach activities where students can come together to learn about harm reductions and to discuss their situations through a peer group setting in a safe space. Another platform can also be online like having a dedicated and regularly staffed Facebook fanpage to offer advice and confidential consultations on sensitive topics like poly-drug use.

## Conclusion

LGBT vocational-school students have a higher prevalence of poly-drug use than their heterosexual peers. LGBT vocational-school students also want counseling, perhaps to assist in dealing with life issues, instead of using drugs. It is thus important for related organizations to provide assistance, advice and other useful ways to help reduce their drug use behaviors.

## Data Availability

The datasets used and analyzed in this study are available from the corresponding author upon reasonable request.

## Abbreviations

GPA: Grade Point Average
LGBT: Lesbian, Gay, Bisexual, Transgender
MSM: Men who have sex with men
